# Genetics and Epigenetics in Allergic Rhinitis

**DOI:** 10.3390/genes12122004

**Published:** 2021-12-17

**Authors:** Bo Yoon Choi, Munsoo Han, Ji Won Kwak, Tae Hoon Kim

**Affiliations:** Department of Otorhinolaryngology-Head & Neck Surgery, College of Medicine, Korea University, Seoul 02841, Korea; bychoi0810@gmail.com (B.Y.C.); mshan35@gmail.com (M.H.); jwon1111@naver.com (J.W.K.)

**Keywords:** allergic rhinitis, allergy, genetics, epigenetics, GWAS, asthma

## Abstract

The pathogenesis of allergic rhinitis is associated with genetic, environmental, and epigenetic factors. Genotyping of single nucleotide polymorphisms (SNPs) is an advanced technique in the field of molecular genetics that is closely correlated with genome-wide association studies (GWASs) in large population groups with allergic diseases. Many recent studies have paid attention to the role of epigenetics, including alteration of DNA methylation, histone acetylation, and miRNA levels in the pathogenesis of allergic rhinitis. In this review article, genetics and epigenetics of allergic rhinitis, including information regarding functions and significance of previously known and newly-discovered genes, are summarized. Directions for future genetic and epigenetic studies of allergic rhinitis are also proposed.

## 1. Introduction

The prevalence of allergic rhinitis (AR) has been on the increase worldwide. The frequency of hypersensitive reactions to multiple allergens has increased. Over 40% of populations in the United States and Europe suffer from allergies [1]. AR contributes to nonproductive time at work and school, reduced participation in outdoor activities, and sleep problems in affected children. AR greatly increases the likelihood of asthma. More than 40% of patients with AR have accompanying asthma [2].

AR is characterized by sneezing, nasal itching, airflow obstruction, and watery rhinorrhea resulting from IgE-mediated responses to inhaled allergens and type 2 helper T (Th2) cell-induced mucosal inflammation [3]. It is closely related to other inflammatory diseases that affect respiratory mucous membranes, such as asthma and allergic conjunctivitis. AR is the result of a complex immunologic response to allergens [4]. At the beginning of sensitization, antigen-presenting cells uptake an allergen at a mucosal site, inducing activation of antigen-specific T cells at draining lymph nodes. Simultaneous activation of epithelial cells then induces the release of epithelial cytokines such as IL-25, IL-33, and thymic stromal lymphopoietin. This process affects a Th2 cell response, which is directed to dendritic cells, causing Th2 innate lymphoid cells and basophils to release cytokines such as IL-13 and IL-4. Such release results in the production of Th2 cells, which can turn B-cells into plasma cells to produce allergen-specific IgE antibodies. Upon re-exposure, the allergen attaches to IgE on the surface of mast cells and circulating basophils, resulting in activation of those cells and the release of histamine and leukotrienes [5,6]. These mediators can cause typical symptoms of AR. In addition, local activation of Th2 lymphocytes results in the release of cytokines that coordinate the entry of inflammatory cells to the mucosa, making nasal mucosa more sensitive to allergens. Histamine released by mast cells can activate blood vessels to increase vascular permeability, while leukotrienes cause vasodilatation. A variety of central reflexes are triggered by activation of sensory nerves, including a motor reflex that stimulates sneezing and parasympathetic reflexes that cause nasal discharge and vasodilatation. In addition, the sympathetic nerve to venous sinusoids is inhibited, leading to vascular congestion and nasal obstruction [7,8].

**Figure 1:**
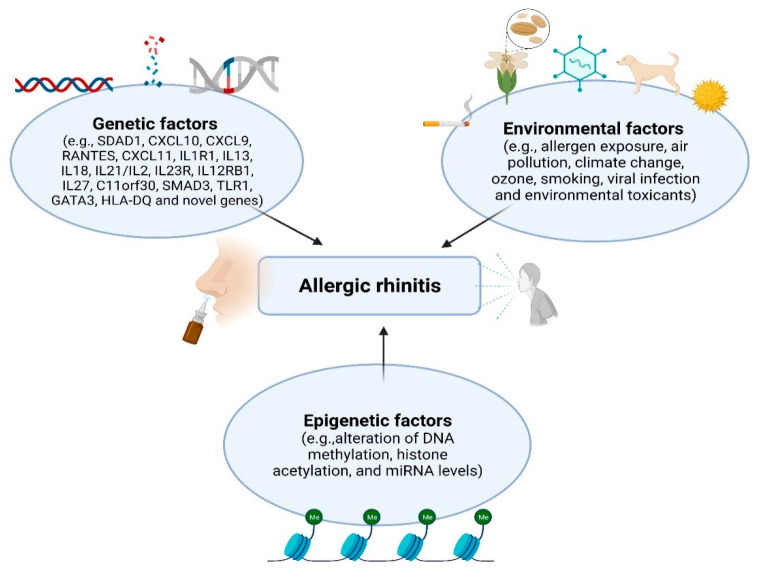
Environmental/genetic/epigenetic factors in AR (Figure was created with Biorender.com, accessed on 13 December 2021).

The pathogenesis of AR and other allergic diseases is complicated. Associations with genetic and environmental factors are known to be involved in these immunologic situations (Figure 1). Environmental determinants, such as allergen exposure, air pollution, climate change, ozone, smoking, viral infection, and environmental toxicants, may underlie much of the increase in AR prevalence. In addition, specific epigenetic changes caused by environmental exposure may contribute to cellular homeostasis and the development of allergic diseases.

Many recent studies have paid attention to the importance of epigenetics [9]. It is accepted nowadays that changes in gene functions without changes in DNA sequences are important in the pathophysiology of many diseases [10]. We have focused on the study of enzymes and processes controlling and managing diseases associated with epigenetic changes [11]. Studies have elucidated the role of epigenetics, including alteration of DNA methylation, histone acetylation, and miRNA levels, in the pathogenesis of AR [12]. In host cells, many changes to DNA methylation [13] and histone acetylation [14] can occur in response to allergens. For allergic rhinitis, it has been recently informed that the severity of allergic responses can be predicted by the DNA methylation level in the *SLFN12* gene when exposed to grass pollen [15]. Histone modification and alterations in miRNA level have been demonstrated to be different at candidate gene level in patients with AR [12].

With the advance of biomedical techniques in the field of molecular genetic science, approaches to elucidating the genetics of allergic diseases have also evolved. In 2002, genome-wide genotyping was introduced, allowing genotyping of hundreds of thousands of single nucleotide polymorphisms (SNPs) and simple variants, such as insertions/deletions and repeats. The opportunity to genotype markers has increased discernment of the genetic structure of humans, revealing that SNPs are closely correlated with each other [16]. This has enabled genome-wide association studies (GWAS) in large groups of the population with allergic disease, allowing researchers to extend the scope of studies concerning the genetics of allergic disease beyond linking it with genetic association studies [17].

Seven loci significantly associated with AR have been identified through genome-wide association studies (GWAS). Many other loci have also been identified through GWAS of related allergic diseases, such as asthma, atopic dermatitis, and allergic sensitization [18,19,20,21]. Recently, with increasing interest in genetics, many genetic studies have been conducted. However, few have focused on the genetics of AR. The purpose of this review article was to summarize current studies surrounding the genetics and epigenetics of AR. In addition, we outlined future directions for research of AR.

## 2. Heredity in AR

### Twin Studies

AR is one of the most common atopic diseases worldwide. The heritability of AR has been estimated to be over 0.65, indicating a strong genetic component [22]. Twin studies open up the possibility of the importance of genetic factors and provide strong evidence for atopic disease. The concordance rate for atopic dermatitis in identical twins is approximately 80%, which is much higher than the 20% concordance rate observed in fraternal twins [23]. Heritability estimates for AR are approximately 91% [24]. AR, atopic dermatitis, and asthma are clinically distinct diseases. However, there is strong evidence that they are correlated, with correlation estimates of 0.55 for asthma and atopic dermatitis, 0.47 for asthma and AR, and 0.62 for atopic dermatitis and AR [25]. Similarity between phenotypes of atopic disorders indicates that biological and etiological factors may also overlap between these disorders. Common genetic risk variants that affect many atopic disorders imply that they can demonstrate common pathogenic characteristics and provide ideas for the development of new treatments. Preschoolers who are not treated for AR have a three-fold increased risk of asthma in adulthood [26]. Similar to other allergic diseases, twin studies offer evidence for the genetic heritability of AR [27]. The concordance rate for identical twins is 45% to 60%, higher than that for fraternal twins of no more than 25%. The calculated heritability of AR is between 33% and 91% [25].

## 3. Overlapping Genetic Architecture in AR and Asthma

### 3.1. Genetics of Asthma

Asthma concordance for identical twins is about 50% [28]. The heritability of asthma is 0.40–0.85 [29]. In 2007, a GWAS of asthma was first published [30]. Gene loci on chromosome 17q12-21, including *ORMDL3/GSDML*, *IKZF3*, and *ZPBP2*, have been reported to be associated with childhood asthma [31]. Subsequent studies demonstrated that *ORMDL3/GSDML* variants are associated with a high risk of asthma in diverse ethnic groups [32,33,34,35]. These associated variants are associated with mRNA expression of *ORMDL3* in lymphoblastoid cell lines. Another large-scale GWAS for asthma has identified six asthma-susceptible loci, including *IL1RL1/IL18R1, SMAD3, ORMDL3/GSDM, IL-33, IL2RB*, and *HLA-DQ* [33]. In particular, several GWAS for asthma have reported the involvement of the IL-33–IL-1RL1 receptor pathway, with *CDHR3* and *ORMDL3* being the loci associated with induced eosinophilia and Th2 inflammatory or viral responses [20,36]. Thymic stromal lymphopoietin (TSLP) and variants near TSLP involved in Th2 inflammation induced by epithelial cell-derived cytokines also show significant associations with asthma [18,36].

For asthma, several asthma-associated loci, including *IL1R1, IL1RL1, IL13, SLC25A46, IL18R1, GSDMB, SMAD3*, and HLA regions can influence allergic sensitization [19]. In both asthma and allergic diseases, six index SNPs are known to represent genome-wide significance. Detailed functions of these SNPs are described below. *SLC25A46* and *TMEM232* encode transmembrane proteins involved in solute transport. *SMAD3* is related to inflammation progression due to immune responses of T-helper 2 (Th2) cells and immune-related cytokines [37]. *C11orf30* is involved in increased total serum IgE levels, with higher susceptibility to poly-sensitization [38,39]. *D2HGDH* and *GAL3ST2* encode D-2 hydroxyglutarate dehydrogenase enzyme-related metabolism and tumor metastasis-related galactose-3-O-sulfotransferase 2 enzyme, respectively [40,41].

Fine-mapping of IgE-associated loci 1q23, 5q31, and 12q13 confirmed the associations of SNPs in 1q23, 5q31, and 12q13 with IgE regulation. For 1q23 and 5q31, the majority were associated with mild to moderately elevated IgE levels, while in the 12q13 locus, single-nucleotide polymorphisms (SNPs) were found to strongly elevate IgE levels. In 1q23, 5q31, and 12q13 loci, SNPs were found to correlate specifically with atopic asthma [42].

Genetic association studies have identified about a total of 50 genes, many of which have been identified by linkage and gene mapping. Effect estimates are measured as odds ratios ranging from 0.5 to 1.5. These gene variants can predict less than 10% of the heritability of asthma [43].

### 3.2. Overlapping Genes in AR and Asthma

**Figure 2:**
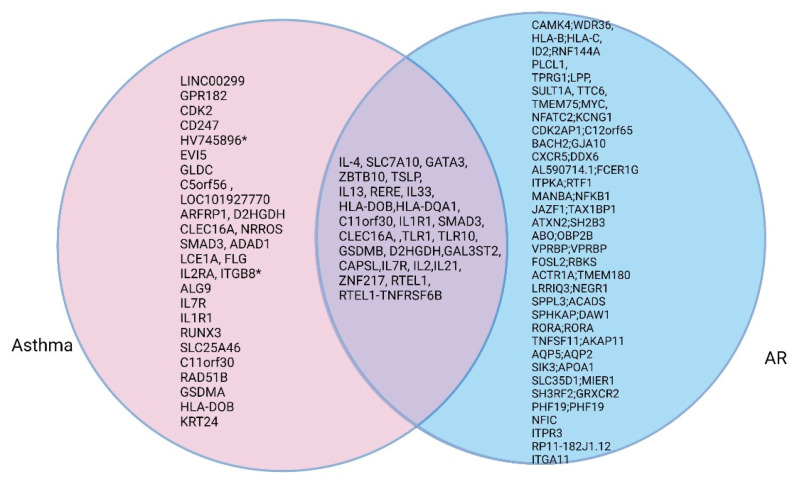
Venn diagram of genes shared between patients AR and asthma.

The concept of united airway disease appears to be due to the close relationship and overlapping genetic characteristics involving AR and asthma. The pathogenesis that involves respiratory epithelial cells and the inflammatory response of AR and asthma are similar [44]. Although genetic susceptibility to allergic diseases is shared, heterogenic features for defining allergy phenotypes are unique to each disease. There are strong genetic associations between AR and asthma or allergic sensitization or eczema, as well as specific shared loci among allergic diseases. To understand the strong associations between AR and asthma in terms of their genetic etiology, we identified overlapping genes by listing representative genes in asthma and AR. Figure 2 presents such representative genes and overlapping genes in patients with asthma or AR. Among the top-ranked genes, 25 independent representative loci were related to both diseases (Figure 2).

Genes and cytokines including IL3, IL4, IL5, IL13, IL33, and GM-CSF are immune-related and involved in susceptibility to AR [45]. *C11orf30/LRRC32* on chromosome 11 is a significant locus associated with both AR and asthma [20,46]. Exposure to an allergen allows immune-related cells to release IgE and causes local tissues to release inflammatory mediators such as IL-4 and IL-13 [47,48].

In a GWAS study, eleven significant variants associated with the risk of asthma with rhinitis were identified, of which *ZBTN10* and *CLEC16A* were significant [20]. A genetic variant of nuclear factor I/A(*NFIA*) gene in the 1p31 chromosomal region is significantly associated with combined asthma and AR phenotype [49,50]. The linkage at chromosomal locus of 5q31.1 consists of the loci at *IL4-KIF3A* in patients with AR and asthma [51,52].

Most of the shared genes are associated with inflammatory processes. Related genes or cytokines, including IL4, IL4R, IL5, IL13, TSLP, IGHG4, and FCER2 are involved in Th2 cell immune response with specific functions. FCER2, expressed in macrophages, eosinophils, B cells, and platelets, regulates IgE responses and is involved in antigen presentation, differentiation of T and B cells, and cellular adherence [53,54]. It is known that CCL26, CCL11, RNases, and IL5 function in recruiting eosinophil in allergic diseases. IL-10 and FOXP3 are involved in important function of regulatory T cells. Especially, IL-10 is a B cell immunosuppressive cytokine with anti-inflammatory response [55]. IL-5 plays an important role in development and function of eosinophil in asthma patients. It has been treated as a promising target currently [56]. IFNG and IL17A are related to TH1 and TH17 cells, respectively. TSLP produced in epithelial cells is involved in the innate immune response to inflammation induced by Th2 cell. IL-4 is an important mediator in allergic airway disease [57]. Systematic reviews have indicated that most loci are related to chromosomal regions, including chromosomes 6p21, 5q31-32, 11q13, and 12q14-24 [58]. The HLA region on chromosome 6q21 are the most significant loci. In studies of classical HLA alleles and amino acid variants to analyze the number of associations at immunological significant locus, *HLA-B* and *HLA-DQB1* were identified as the strongest associated HLA class genes [59]. In GWAS of allergic sensitization and non-allergic rhinitis, the most significant loci highly overlapped with those of AR. These alterations occur within the peptide binding pocket. They are related to changes in antigen binding properties, especially antigen presentation. According to results of GWAS of asthma and allergic disease, HLA-associated with allergic disease is different from HLA-associated with asthma [60].

## 4. GWAS in AR

### 4.1. Previously Known Loci in AR

In AR studies, many genes are involved in diverse immune-related diseases, including allergic and autoimmune disorders. These genes include *SDAD1, CXCL10, CXCL9, RANTES, CXCL11, IL1R1, IL13, IL18, IL21/IL2, IL23R, IL12RB1, IL27, C11orf30, SMAD3, TLR1, GATA3*, and *HLA-DQ*.

*BCAP* on chromosome 10q24.1 and *MRPL4* on chromosome 19p13.2 were commonly associated with AR and atopy in a GWAS of a Chinese cohort [61]. Linkages within known regions, such as *HLA-DQ* and *NPSR1* loci, were also replicated. The 19p13.2 locus regulates allergic reactions by affecting soluble intercellular adhesion molecule 1 [62]. SNPs in the *TNF-α* gene are also known as a high risk factor of AR [63]. A large scale GWAS meta-analysis has indicated that *C11orf30* and *LRRC32* have genome-wide significance. *C11orf30* encodes a protein with epithelial barrier function [64]. *LRRC32* encodes a surface receptor of T cell for latent TGF-β and plays an important role in immune tolerance by regulatory T-cell function [65]. These two loci have been previously identified in GWAS of both asthma and atopic dermatitis [20,46]. A locus near *FERD3L* on chromosome 7p21.1 is significant in a genome-wide meta-analysis for all ethnic populations [21].

### 4.2. Novel Loci Identified in AR

Recently the largest GWAS has found 41 significant risk loci of AR, including 20 novel loci [59]. Most of these novel loci have functions in innate and adaptive immune processes. These include *IL7R* at 5p13.2 that is previously associated with eczema [66] and involved in V(D)J recombination of T- and B-cell receptors for cellular activation by the Th2 immune inducer TSLP [67]. *SH2B3* on chromosomal 12q24.12 is related to blood eosinophil count [68] and inflammatory pathways by regulating T cell activation [69]. A locus on chromosomal 19q13.11 and *CEBPA* have been previously linked to eosinophilic esophagitis [70] and associated with myeloid cell lineage differentiation [71], respectively. In addition, *CEBPG* plays a role in an enhancer binding protein for IGH, IL4, IL6, and IL8 [72].

Most of the additional novel loci have known immune-related characteristics. A locus at 11q23 near *CXCR5* encodes a B-cell chemokine receptor and follicular T-cells. It functions in the migration of B-cells and helps interactions between B- and T-cells within lymph nodes [73]. *FCER1G* at chromosomal 1q23.3 encodes γ chain of IgE receptor, which is an essential component of allergic responses. *NFKB1* at 4q24 encodes a subunit of the NFκB complex by functioning in the activation of inflammatory pathways [74]. *BACH2* at 6q15 plays an immune-modulating role in the production of memory B- and T- cells induced by antigen [75,76]. *LTK* and *TYRO3* at 15q15.1 can regulate Th2 immunity and TLR signaling, respectively. *SPPL3* and *OASL* at 12q24.31 are required for mature NK cell and IFN-αsignaling, respectively. *RORA* at 15q22.2 plays a role in the development of Th2 innate lymphoid cell and inflammation process. *TNFSF11* at 13q14.11 functions in T-cell activation by dendritic cells. *VPRBP* at 3p21.2 has roles in T-cell proliferation after antigen recognition. It is involved in V(D)J recombination during B-cell development [77,78,79,80,81].

Table 1 summarizes the function, mechanism, and chromosomal locations of representative genes of previously known and novel genes identified in AR.

## 5. Epigenetics in Allergic Rhinitis

Studies conducted on patients with AR and immune cells isolated from patients with AR have shown that histone deacetylase (HDAC) is increased in immune cells and that inhibition of HDAC can help improve the condition of AR. A study of patients with AR has shown that HDAC1 is upregulated in the nasal epithelial cells compared to that in healthy controls [86]. Interleukin-4 (IL-4) can increase the expression of HDAC1, producing nasal epithelial barrier dysfunction [87]. HDAC1 inhibitors such as trichostatin A and sodium butyrate can inhibit nasal epithelial dysfunction in mice [87]. Many studies have reported that the expression of TWIK-related potassium channel-1 (TREK-1) is significantly down-regulated in patients with AR [47,48,53]. TREK1 expression is up-regulated and HDAC1 is down-regulated in the nasal mucosa by antigen-specific immunotherapy [86]. Thus, increased expression of HDAC in nasal epithelial cells may reduce the expression of TREK1, producing inhibitory effects in AR. Inhibition of HDAC1 promotes IL-10 and Foxp3, a main regulator in regulatory T cell functions, and blocks excessive activation of immune cells [88]. HDAC inhibitors may decrease the expression of TNF-α [89]. These findings indicate that an increase in HDAC activity might contribute to the pathogenesis of AR by increasing pro-inflammatory cytokines and decreasing anti-inflammatory cytokines.

Studies have suggested that changes in DNA methylation might differentiate allergic patients from the healthy people. Patterns of DNA methylation are correlated with patterns and numbers of CD4+ T cells in AR. A study has revealed that allergic children have changes of DNA methylation at CpG sites in blood mononuclear cells and airway epithelial cells [90]. It has been demonstrated that sublingual immunotherapy in patients with respiratory allergy is associated with decreased DNA methylation of CpG sites within the Foxp3 locus in memory regulatory T cells [91]. In addition, hypermethylation of DNA can lead to decreased expression of IFN-γ in patients with AR [92]. The increase in mRNA expression of IL-33 and IgE by DNA hypomethylation has been demonstrated [93]. Alterations in the methylation pattern at the CpG site within the melatonin receptor 1A gene might serve as paternally transmitted genetic variants in AR [94].

MicroRNAs (miRNAs) are small (20–25 nucleotides) non-protein coding RNA molecules that regulate gene expression by cleaving and silencing the target transcript and inhibiting the post-transcriptional process. With the premise that regulation of miRNA-mediated gene expression is one of the epigenetic mechanisms [95], there have been many studies on various miRNAs in AR [96]. One study has demonstrated the correlation between changes in miRNA expression, particularly decreased expression of miR-21 and miR-126 in neonatal mononuclear leukocytes, and the development of AR [97]. In childhood AR, miR-181a levels might be a predictor of disease severity [98]. Changes of miRNAs in patients with AR include upregulation of miR-498, miR-187, miR-874, miR-143, and miR-886-3p and down-regulation of miR-18a, miR-126, let-7e, miR-155, and miR-224 [99].

MiR-29a is highly expressed in the nasal mucosa of AR patients. It can suppress nasal epithelial cell apoptosis and enhance the development of AR by down-regulating FOS expression [100]. Circulating miRNAs have been poorly studied in patients with upper airway diseases, although they have a diagnostic potential for these diseases. A study has confirmed that circulating miR-206, miR-125b, miR-16, miR-126, miR-299-5p, and miR-133b levels can predict allergic and asthmatic status [101]. Endothelial miR-1 levels down-regulated by IL-13 can modulate eosinophil transport in allergic airway inflammation, showing a therapeutic potential in asthma and chronic rhinosinusitis [102].

Other studies have also shown that highly expressed miR-221 and miR-142-3p on nasal mucosa can be biomarkers for AR by promoting mast cell degranulation and reinforcing the degranulation of mast cells, respectively [103,104,105]. Moreover, miR-126-5p is involved in mast cell degranulation. miR-19a-5p and miR-26a-5p are associated with regulation of immunity and pathogenesis of the disease [106,107,108]. Table 2 summarized the functions and targets of AR-specific miRNAs based on researches so far.

## 6. Future Studies

### 6.1. Limitation of Current Genetic Studies in AR

Despite recent advances in GWAS, which may provide more information regarding disease etiology, certain limitations remain in that individual effect and possible interactions have not been resolved. Pinpointing causal SNPs can be challenging due to linkage imbalance, which can span considerably more than one gene. In addition to genetic factors, many other factors can determine treatment responses. Difference in treatment responses between individuals may contribute to the clinical course of AR and treatment responses. They might be caused by diverse factors, such as tissue-specific epigenetic influences and environmental factors, such as smoking and allergen exposure, that can potentially interact with genetic factors. This provides a basis to investigate how eosinophil targeting antibody to novel gene- based cytokines might be correlated with modifications to DNA methylation in eosinophils and pre-treatment epigenetic profiles. Epigenetic profiling could also yield future potential biomarkers that could aid in the classification and selection of patients who would most benefit from potential biological therapies and for whom little improvement could be otherwise expected. Although many target genes involved in AR have been identified recently, how medical science can use this information for the development of novel drugs or repurposing existing drugs remains unclear. Recent findings of genetic variants using genomics-based drug repositioning may provide promising results for AR patients. However, a number of novel AR loci have unknown functions in the pathogenesis of the disorder. Associated loci at 12q24.31 are between *CDK2AP1* and *C12orf65*. Many genes including *CDK2AP1, ABCB9, ARL6IP4, C12orf65, SBNO1, SNRNP35, MPHOSPH9, OGFOD2, PITPNM2, RILPL2*, and *SETD8* function with *DDX55* as enhancer-promoter in various immune cells of blood and lung tissues. However, none of these genes has any obvious AR-related features. Many other related genes, such as *NEGR1* at 1p31.1, *RERE* at 1p36.32, *FOSL2* at 2p23.2, and *JAZF1* at 7p15.1, are similar in blood and lung tissues [59]. Future studies regarding these genes and loci will be helpful and necessary to investigate AR pathogenesis and find novel drug targets. It is required to find out the link between SNPs and gene expression by identifying related cell types expressing the gene and confirming mRNA translation. 

### 6.2. Directions for Future Studies

Currently main pharmacotherapy for AR includes oral/nasal anti-histamines, intranasal corticosteroids, leukotriene antagonists, anti-cholinergics, anti-IgE monoclonal antibodies, and mast cell stabilizers. These medications mainly aim at reducing symptoms related to AR. Limitations of these currently used pharmacotherapies are that the lasting time of effects is short and symptoms begin to appear when stopping medications [120]. Among current treatments, allergen-specific immunotherapy in which allergens are administered repeatedly subcutaneously or sublingually shows relatively long term effects in severe AR patients [121]. However, current immunotherapy has a disadvantage in that it should be administered for more than two years in order to show clinically visible effects [122,123]. There is a need to investigate other appropriate medication targets for efficient and long-term management of AR. Researches have indicated that epithelial cell-derived cytokines that regulate T cell activation in allergic diseases [124] and targeting specific cytokines and T cells could also represent potential benefits in patients with AR. 

Epigenetic studies began with the goal of controlling the long term production of cytokines and T cell proliferation [125]. It has been studied as a potential drug target to suppress changes in the imbalance of T cell and cytokine profile by normalizing gene expression in AR. Studies have shown that long-term effects of immunotherapy are associated with long lasting effects of epigenetic changes [126] and may be secondary to epigenetic changes [91,127]. The generation of antigen-specific regulatory T cells (Treg) is required to induce and maintain tolerance to antigen. Demethylation of Treg-specific demethylated region (TSDR) in FOXP3 is essential for the maintenance of the inhibitory properties of Tregs [128,129]. Despite a low number of people analyzed in two studies, demethylation of FOXP3 can be a promising marker for the induction of tolerance. Analysis with a mouse model of AR has revealed that dosing synthetic anti-inflammatory miRNA concurrently with immunotherapy may improve the result of only immunotherapy [130]. It also suggests that demethylation of FOXP3 in AR may be a necessary factor for successful immunotherapy. It may play a role in predicting the response of immunotherapy. Studies investigating biomarkers for the response to omalizumab, a monoclonal antibody against IgE, which is currently marketed as a treatment for AR, has not been published so far. No study has reported genetic susceptibility of dupilumab, the latest monoclonal antibody against the receptor chain by IL-4 and IL-13. Genetics can aid in genetic determinants that may contribute to responses to specific biologicals. Epigenetic studies that predict the outcomes of biologicals are also currently scarce. It may be promising to investigate associations of the degree of methylation with associated gene and the response to a monoclonal antibody currently in use.

Epigenetic controls function as resistance to self-antigens, regulating immune responses. Epigenetic dysregulations can lead to immune disorders [131]. An epigenetic study has performed methylated DNA sequencing of lung tissue DNA from mice exposed to house dust mite (HDM) and found that continued exposure to HDM can cause airway inflammation and hypersensitivity by extensive changes in methylation and hydroxymethylation of specific genes, including *SMAD3* and *TGFβ2* [132]. These findings indicate that exposure to allergens could trigger epigenetic changes.

Although still in its infancy, allergic disease-associated SNPs are linked to potential drug targets. Main SNPs were associated with several gene transcripts. It may be due to functional regulation of several genes by one SNP or disequilibrium at a high linkage level. Coding mutations can alter amino acid sequences, resulting in changes in protein function. These mutations result in protein truncation or production of different amino acids by mRNA splicing [133]. Changes in amino acid levels affect protein altering, leading to changes of protein function, shape, and consequently protein concentrations. So far, few allergic disease loci have been studied for functional validation linking genetic risk and mechanism of disease. Precise mapping studies are needed to pinpoint variants and relevant genes for the development of SNPs targeting drug.

With the rapid development of genetics, we can present new drug targets and potential drug repositioning based on genomics for the therapy of AR. Drug repositioning means identifying drugs that are available in the market or in development, and repurposing them for specific uses. This method increases the efficiency of drugs and reduces time and cost due to the established safety framework [134].

GWAS can identify disease associated genes, providing a powerful technique for validating existing targets and uncovering new drug targets. Genes that encode proteins targeted by drugs already on the market have been identified by a number of genetic studies. For instance, a GWAS for dyslipidemia has revealed that the *HMGCR* gene that encodes the HMG-CoA reductase protein is a target for statins used to lower blood cholesterol levels. This finding is significant in that gene-targeting drugs discovered by genetic studies are more than twice as successful as drugs without genetic basis in reaching the market [135]. Although there is no case of a genomics-based drug in the respiratory or allergy field yet, it may offer potentials. Genetics can also detect targeted genetic variants with protective functions, such as a genetic variant in IL33, one of the common genes of asthma and AR. This variant reduces the expression of the proteins and reduces receptor binding, which in turn can lower circulating blood eosinophils and decrease the risk of asthma development [136]. Individuals with this genetic variant tend to be at lower risk for other allergic diseases, showing that lowering IL-33 levels or partially blocking IL-33 function might be functionally useful. 

**Figure 3:**
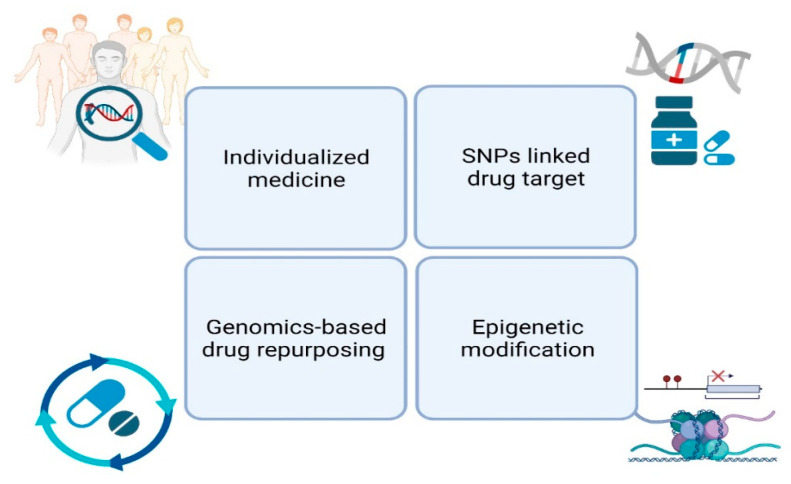
Directions for future studies.

Pharmacogenetics is a promising topic in the field of allergy genetics. Among them, individualized medicine is sought-after the most to identify medications that should be used for specific patients. In patients with severe allergic disease who do not respond to standard therapy, the lack of appropriate management is the main cause of treatment failure. The aim of genetic researches for allergic disease is to achieve improved therapeutic outcomes and minimize side effects by tailoring individualized therapy to specific genotypes. Using genetics, it will be possible to identify and early discriminate patients who are difficult to treat disease, allowing them to proceed with novel personalized treatments. Personalized treatment options in which specific, targeted genetic modifications are implemented are likely to play important roles in monitoring or predicting outcomes for personalized therapy (Figure 3).

## 7. Conclusions

In this study, we reviewed the current status of genetic and epigenetic studies. Through expression quantitative trait locus (eQTL) mapping and coding sequence variation, we provided the latest genetic updates concerning GWAS information and AR, with an emphasis on genetic functions. Although research involving AR and asthma has made great progress to date, there remain many unexplored areas of allergic diseases among GWAS studies [137]. Many studies have been conducted on overlapping genetic significant loci and possible linked pathways among allergic disorders. However, it is difficult to differentiate between disease-specific and shared variants due to the comorbidity of multiple allergic diseases [138]. Thus, more studies are needed to understand underlying mechanisms and linkage of atopic diseases. 

Genetic associations do not function equally to biological functions. Consequently, research is necessary to apply initial findings to biological insights that will further accelerate development of clinical diagnostic techniques and therapeutic applications. The final purpose of our research regarding genes implicated in AR and large scale GWAS studies on AR is to use the information of novel identified variants for diagnosis and treatment in the clinic. In the near future, using individual whole-genome sequencing to identify individual genetically at-risk patients and administer individualized medicine seems to be feasible. Future research might also include detailed genotyping of diverse ethnic populations, improving our understanding of environmental and epigenetic factors, and applying new tools using genome sequencing, epigenetics in specific tissues, and a systemic biologic approach. Systemic biology includes the aggregation of big data for integrated profiles of disease, providing better understanding of pathogenesis and disease outcome [139]. It involves the exchange of biological expression from genotype to phenotype using advanced systemic and mathematical methods [140]. Using systemized process to analyze patterns in a systematic collection of large molecular data and clinical data, can support individualized therapies for allergic diseases with similar subtypes and provide clinical insights into the pathogenesis of allergic diseases [141].

Many current GWAS studies tend to be limited to specific ethnic groups, especially Europeans. Thus, whether GWAS results can be applied more broadly to various ethnic groups remains unclear. Designing genetic studies that include racially and ethnically diverse participants with allergic diseases is needed in the future [142].

## Figures and Tables

**Table 1:** Summary of genomic loci of allergic rhinitis.

Chromosome	Gene	Possible Allergic Mechanism	References
19p13	*MRPL4*	Involved in inflammatory adhesion process.	[61]
10q24	*BCAP*	B cells development, activation, and maturation.	[61]
11q13	*C11orf30* */* *LRRC32*	Epithelial barrier function, regulatory	[18]
		T-cell function, and immune tolerance.	
4p14	*TLR6*	Pattern recognition receptors in innate immunity.	[18]
4q27	*IL2*	Immune regulatory effects.	[19]
5q22.1	*TSLP*	Th2 immune responses.	[18]
	*TMEM232, SLCA25A46*		
6p21.32	*HLA region (* *BTNL2* *,* *HLA-DPB1* *,* *HLA-DQA1* *,* *HLA-DQB1* *,* *HLA-DRB1* *,*	Antigen presentation, self tolerance.	[18]
	*HLA-DRB3,HLA-DRB4,HLA-DRB5,TAP1, andTAP2*)		
11q13.5	*C11orf30* *, LRRC32*	Expressed in regulatory T cell, involved in TGF-β signaling.	[18]
14q23.1	*PPM1A, DHRS7*		[18]
16p13.13	*CLEC16A*	Highly expressed in lung, T- and B-cells with unknown function.	[20]
5p13.2	*CAPSL; IL7R*	V(D)J recombination of B and T cell receptors.	[59]
		T cell subtypes have different levels of IL-7R on the cell surface.	
12q24.31	*CDK2AP1; C12orf65*	Involved in downstream of T cell receptor activation. Involved in hematopoiesis.	[59]
6q15	*BACH2; GJA10*	Production of memory T cells and memory B cells induced by antigen.	[59]
11q23.3	*CXCR5; DDX6*	Chemokine receptor expressed on B cells and involved in B cell migration in spleen and lymph nodes.	[59]
		CXCR5 is expressed on a subset of follicular T cells.	
1q23.3	*AL590714.1; FCER1G*	Encodes IgE receptor in epithelial cells and immune cells. Tanb	[61]
15q15.1	*ITPKA; RTF1*	TYRO3 inhibits immune signaling mediated from TLR and activates SOCS1. Leukocyte tyrosine kinase is involved in downstream of T cell receptor activation.	[59]
4q24	*MANBA; NFKB1*	NFKB1 is involved in activation of inflammatory pathways, mediating signals from TLRs and cytokines.	[82]
7p15.1	*JAZF1; TAX1BP1*	Transcriptional repressor in endometrial stromal tumors, multiple sclerosis and Th2 diabetes.	[59]
12q24.12	*ATXN2; SH2B3*	Involved in hematopoiesis and downstream of T cell receptor activation.	[59]
9q34.2	*ABO; OBP2B*	Allele variants of ABO determine blood group type.	[59]
3p21.2	*VPRBP*	Involved in T cell proliferation and V(D)J recombination in B cell development.	[59]
2p23.2	*FOSL2; RBKS*	Cell cycle and proliferation.	[59]
10q24.32	*ACTR1A; TMEM180*	Subunit of NFKB complex and involved in regulating TLR-4 and cytokine signaling.	[59]
1p31.1	*LRRIQ3; NEGR1*	Cell adhesion.	[59]
19q13.11	*CEBPA; SLC7A10*	CEBPG is involved in transcriptional enhancers for the immunoglobulin heavy chain. CEBPA is involved in lung development and inflammatory bowel disease.	[83]
12q24.31	*SPPL3; ACADS*	Regulate the number of NK cells. OASL is involved in IFN-γ signaling.	[59]
2q36.3	*SPHKAP; DAW1*	Dynein assembly factor.	[59]
15q22.2	*RORA*	Involved in development of natural helper cell.	[84]
1p36.23	*RERE; SLC45A1*	Apoptosis-associated transcription factor.	[85]
13q14.11	*TNFSF11; AKAP11*	Help dendritic cells to enhance T cell activation.	[59]

**Table 2:** Summary of miRNAs in allergic rhinitis.

miRNA	Target	Function	References
let-7	JAK1/STAT3, IL-13, SOCS4	Regulating IL-13 secretion and modulating Th2 inflammation.	[99,109,110]
miR-206	S100A7A, VEGF	Involved in the VEGF pathway	[101]
miR-338-3p	WNT/ β-Catenin	inducing epithelial-mesenchymal transition by inhibition of Wnt/β-catenin pathway.	[101]
miR-16	IκB/NF-κB	Prevents IL-13-induced inflammatory cytokine secretion.	[111]
miR-498	STAT3	Suppressing Th17 cell differentiation.	[99,101]
miR-187	CD276	Regulation of T-cell response.	[99,112]
miR-143	TGF-β1	Modulates memory T-cell differentiation.	[99,112]
miR-886-3p	SMAD3, FoxO1	Regulates TGF pathway via SMAD3.	[99,112]
miR-224	SMAD4	Regulates TGF pathway via SMAD4.	[99,112]
miR-155	IL13Ra1	Control the proliferation and differentiation of Treg cells and regulates IL-13 pathway in macrophages.	[113,114,115,116]
miR-126	VEGF, IRS1	Decreased expression in mononuclear leukocytes, regulating IL4 effect.	[117]
miR18a	CTGF	Involved in TGF pathway.	[101]
miR-205	MICAL2	Activation of ERK17 pathway.	[99]
miR-375	JAK2/STAT3	Prevents apoptosis of nasal mucosa cells	[118]
miR-19a	TGFβ1	attenuate allergen-induced suppression of IL-10 in the peripheral dendritic cells.	[117]
miR-26a	SMAD2, SMAD3	Modulation of TGF-β-dependent signaling pathways and repression of inflammatory responses by promoting regulatory T-cell responses or through NF-κB inhibition.	[101]
miR-135a	GATA-3	Increase in the levels of IL-4 and IgE in nasal mucosa, prevent mast cell activation/degranulation.	[119]

## Data Availability

Not Applicable.

## References

[1] Salo P.M., Arbes S.J., Jaramillo R., Calatroni A., Weir C.H., Sever M.L., Hoppin J.A., Rose K.M., Liu A.H., Gergen P.J. (2014). Prevalence of allergic sensitization in the United States: Results from the National Health and Nutrition Examination Survey (NHANES) 2005–2006. J. Allergy Clin. Immunol..

[2] Shaaban R., Zureik M., Soussan D., Neukirch C., Heinrich J., Sunyer J., Wjst M., Cerveri I., Pin I., Bousquet J. (2008). Rhinitis and onset of asthma: A longitudinal population-based study. Lancet.

[3] Bousquet J., Khaltaev N., Cruz A.A., Denburg J., Fokkens W.J., Togias A., Zuberbier T., Baena-Cagnani C.E., Canonica G.W., van Weel C. (2008). Allergic Rhinitis and its Impact on Asthma (ARIA) 2008 update (in collaboration with the World Health Organization, GA(2)LEN and AllerGen). Allergy.

[4] Wheatley L.M., Togias A. (2015). Clinical practice. Allergic rhinitis. N. Engl. J. Med..

[5] Barnes P.J. (2011). Pathophysiology of allergic inflammation. Immunol. Rev..

[6] Sin B., Togias A. (2011). Pathophysiology of allergic and nonallergic rhinitis. Proc. Am. Thorac. Soc..

[7] Sarin S., Undem B., Sanico A., Togias A. (2006). The role of the nervous system in rhinitis. J. Allergy Clin. Immunol..

[8] Wachs M., Proud D., Lichtenstein L.M., Kagey-Sobotka A., Norman P.S., Naclerio R.M. (1989). Observations on the pathogenesis of nasal priming. J. Allergy Clin. Immunol..

[9] Huang B., Jiang C., Zhang R. (2014). Epigenetics: The language of the cell?. Epigenomics.

[10] van der Harst P., de Windt L.J., Chambers J.C. (2017). Translational Perspective on Epigenetics in Cardiovascular Disease. J. Am. Coll. Cardiol..

[11] Dong G., Chen W., Wang X., Yang X., Xu T., Wang P., Zhang W., Rao Y., Miao C., Sheng C. (2017). Small Molecule Inhibitors Simultaneously Targeting Cancer Metabolism and Epigenetics: Discovery of Novel Nicotinamide Phosphoribosyltransferase (NAMPT) and Histone Deacetylase (HDAC) Dual Inhibitors. J. Med. Chem..

[12] North M.L., Ellis A.K. (2011). The role of epigenetics in the developmental origins of allergic disease. Ann. Allergy Asthma Immunol..

[13] Gao Z., Huang M., Qu Z., Wang J., Cai X. (2019). Identification of DNA methylation module in seasonal allergic rhinitis. Int. J. Pediatr. Otorhinolaryngol..

[14] Wang J., Wen L., Wang Y., Chen F. (2016). Therapeutic Effect of Histone Deacetylase Inhibitor, Sodium Butyrate, on Allergic Rhinitis In Vivo. DNA Cell Biol..

[15] North M.L., Jones M.J., MacIsaac J.L., Morin A.M., Steacy L.M., Gregor A., Kobor M.S., Ellis A.K. (2018). Blood and nasal epigenetics correlate with allergic rhinitis symptom development in the environmental exposure unit. Allergy.

[16] Ozaki K., Ohnishi Y., Iida A., Sekine A., Yamada R., Tsunoda T., Sato H., Sato H., Hori M., Nakamura Y. (2002). Functional SNPs in the lymphotoxin-α gene that are associated with susceptibility to myocardial infarction. Nat. Genet..

[17] Power R.A., Parkhill J., de Oliveira T. (2017). Microbial genome-wide association studies: Lessons from human GWAS. Nat. Rev. Genet..

[18] Ramasamy A., Curjuric I., Coin L.J., Kumar A., McArdle W.L., Imboden M., Leynaert B., Kogevinas M., Schmid-Grendelmeier P., Pekkanen J. (2011). A genome-wide meta-analysis of genetic variants associated with allergic rhinitis and grass sensitization and their interaction with birth order. J. Allergy Clin. Immunol..

[19] Bonnelykke K., Matheson M.C., Pers T.H., Granell R., Strachan D.P., Alves A.C., Linneberg A., Curtin J.A., Warrington N.M., Standl M. (2013). Meta-analysis of genome-wide association studies identifies ten loci influencing allergic sensitization. Nat. Genet..

[20] Ferreira M.A., Matheson M.C., Tang C.S., Granell R., Ang W., Hui J., Kiefer A.K., Duffy D.L., Baltic S., Danoy P. (2014). Genome-wide association analysis identifies 11 risk variants associated with the asthma with hay fever phenotype. J. Allergy Clin. Immunol..

[21] Bunyavanich S., Schadt E.E., Himes B.E., Lasky-Su J., Qiu W., Lazarus R., Ziniti J.P., Cohain A., Linderman M., Torgerson D.G. (2014). Integrated genome-wide association, coexpression network, and expression single nucleotide polymorphism analysis identifies novel pathway in allergic rhinitis. BMC Med. Genom..

[22] Greiner A.N., Hellings P.W., Rotiroti G., Scadding G.K. (2011). Allergic rhinitis. Lancet.

[23] Schultz Larsen F. (1993). Atopic dermatitis: A genetic-epidemiologic study in a population-based twin sample. J. Am. Acad. Dermatol..

[24] Ober C., Yao T.C. (2011). The genetics of asthma and allergic disease: A 21st century perspective. Immunol. Rev..

[25] Van Beijsterveldt C.E., Boomsma D.I. (2007). Genetics of parentally reported asthma, eczema and rhinitis in 5-yr-old twins. Eur. Respir. J..

[26] Rochat M.K., Illi S., Ege M.J., Lau S., Keil T., Wahn U., von Mutius E., Multicentre Allergy Study Group (2010). Allergic rhinitis as a predictor for wheezing onset in school-aged children. J. Allergy Clin. Immunol..

[27] Davila I., Mullol J., Ferrer M., Bartra J., del Cuvillo A., Montoro J., Jauregui I., Sastre J., Valero A. (2009). Genetic aspects of allergic rhinitis. J. Investig. Allergol. Clin. Immunol..

[28] Thomsen S.F., van der Sluis S., Kyvik K.O., Skytthe A., Skadhauge L.R., Backer V. (2011). Increase in the heritability of asthma from 1994 to 2003 among adolescent twins. Respir. Med..

[29] Los H., Postmus P.E., Boomsma D.I. (2001). Asthma genetics and intermediate phenotypes: A review from twin studies. Twin Res..

[30] Moffatt M.F., Kabesch M., Liang L., Dixon A.L., Strachan D., Heath S., Depner M., von Berg A., Bufe A., Rietschel E. (2007). Genetic variants regulating ORMDL3 expression contribute to the risk of childhood asthma. Nature.

[31] Bisgaard H., Bonnelykke K., Sleiman P.M., Brasholt M., Chawes B., Kreiner-Moller E., Stage M., Kim C., Tavendale R., Baty F. (2009). Chromosome 17q21 gene variants are associated with asthma and exacerbations but not atopy in early childhood. Am. J. Respir. Crit. Care Med..

[32] Bonnelykke K., Sleiman P., Nielsen K., Kreiner-Moller E., Mercader J.M., Belgrave D., den Dekker H.T., Husby A., Sevelsted A., Faura-Tellez G. (2014). A genome-wide association study identifies CDHR3 as a susceptibility locus for early childhood asthma with severe exacerbations. Nat. Genet..

[33] Moffatt M.F., Gut I.G., Demenais F., Strachan D.P., Bouzigon E., Heath S., von Mutius E., Farrall M., Lathrop M., Cookson W. (2010). A large-scale, consortium-based genomewide association study of asthma. N. Engl. J. Med..

[34] Wan Y.I., Shrine N.R., Soler Artigas M., Wain L.V., Blakey J.D., Moffatt M.F., Bush A., Chung K.F., Cookson W.O., Strachan D.P. (2012). Genome-wide association study to identify genetic determinants of severe asthma. Thorax.

[35] Verlaan D.J., Berlivet S., Hunninghake G.M., Madore A.M., Lariviere M., Moussette S., Grundberg E., Kwan T., Ouimet M., Ge B. (2009). Allele-specific chromatin remodeling in the ZPBP2/GSDMB/ORMDL3 locus associated with the risk of asthma and autoimmune disease. Am. J. Hum. Genet..

[36] Torgerson D.G., Ampleford E.J., Chiu G.Y., Gauderman W.J., Gignoux C.R., Graves P.E., Himes B.E., Levin A.M., Mathias R.A., Hancock D.B. (2011). Meta-analysis of genome-wide association studies of asthma in ethnically diverse North American populations. Nat. Genet..

[37] Anthoni M., Wang G., Leino M.S., Lauerma A.I., Alenius H.T., Wolff H.J. (2007). Smad3-signalling and Th2 cytokines in normal mouse airways and in a mouse model of asthma. Int. J. Biol. Sci..

[38] Li X., Ampleford E.J., Howard T.D., Moore W.C., Li H., Busse W.W., Castro M., Erzurum S.C., Fitzpatrick A.M., Gaston B. (2012). The C11orf30-LRRC32 region is associated with total serum IgE levels in asthmatic patients. J. Allergy Clin. Immunol..

[39] Amaral A.F., Minelli C., Guerra S., Wjst M., Probst-Hensch N., Pin I., Svanes C., Janson C., Heinrich J., Jarvis D.L. (2015). The locus C11orf30 increases susceptibility to poly-sensitization. Allergy.

[40] Struys E.A., Salomons G.S., Achouri Y., Van Schaftingen E., Grosso S., Craigen W.J., Verhoeven N.M., Jakobs C. (2005). Mutations in the D-2-hydroxyglutarate dehydrogenase gene cause D-2-hydroxyglutaric aciduria. Am. J. Hum. Genet..

[41] Seko A., Nagata K., Yonezawa S., Yamashita K. (2002). Down-regulation of Gal 3-O-sulfotransferase-2 (Gal3ST-2) expression in human colonic non-mucinous adenocarcinoma. Jpn. J. Cancer Res..

[42] Sharma V., Michel S., Gaertner V., Franke A., Vogelberg C., von Berg A., Bufe A., Heinzmann A., Laub O., Rietschel E. (2014). Fine-mapping of IgE-associated loci 1q23, 5q31, and 12q13 using 1000 Genomes Project data. Allergy.

[43] Weiss S.T., Silverman E.K. (2011). Pro: Genome-wide association studies (GWAS) in asthma. Am. J. Respir. Crit Care Med..

[44] Morales-Suarez-Varela M., Llopis-Gonzalez A., Gimeno-Clemente N., Jimenez-Lopez M.C., Garcia-Marcos Alvarez L. (2010). International Study of Asthma and Allergy in Childhood Phase III (ISAAC III): The Role of Non-Response in Valencia. Iran. J. Allergy Asthma Immunol..

[45] Lu M.P., Chen R.X., Wang M.L., Zhu X.J., Zhu L.P., Yin M., Zhang Z.D., Cheng L. (2011). Association study on IL4, IL13 and IL4RA polymorphisms in mite-sensitized persistent allergic rhinitis in a Chinese population. PLoS ONE.

[46] Ferreira M.A., Matheson M.C., Duffy D.L., Marks G.B., Hui J., Le Souef P., Danoy P., Baltic S., Nyholt D.R., Jenkins M. (2011). Identification of IL6R and chromosome 11q13.5 as risk loci for asthma. Lancet.

[47] Wynn T.A. (2015). Type 2 cytokines: Mechanisms and therapeutic strategies. Nat. Rev. Immunol..

[48] Barnes P.J. (2001). Th2 cytokines and asthma: An introduction. Respir. Res..

[49] Dizier M.H., Margaritte-Jeannin P., Madore A.M., Moffatt M., Brossard M., Lavielle N., Sarnowski C., Just J., Cookson W., Lathrop M. (2014). The nuclear factor I/A (NFIA) gene is associated with the asthma plus rhinitis phenotype. J. Allergy Clin. Immunol..

[50] Dizier M.H., Bouzigon E., Guilloud-Bataille M., Betard C., Bousquet J., Charpin D., Gormand F., Hochez J., Just J., Lemainque A. (2005). Genome screen in the French EGEA study: Detection of linked regions shared or not shared by allergic rhinitis and asthma. Genes Immun..

[51] Li X., Howard T.D., Zheng S.L., Haselkorn T., Peters S.P., Meyers D.A., Bleecker E.R. (2010). Genome-wide association study of asthma identifies RAD50-IL13 and HLA-DR/DQ regions. J. Allergy Clin. Immunol..

[52] Marenholz I., Esparza-Gordillo J., Ruschendorf F., Bauerfeind A., Strachan D.P., Spycher B.D., Baurecht H., Margaritte-Jeannin P., Saaf A., Kerkhof M. (2015). Meta-analysis identifies seven susceptibility loci involved in the atopic march. Nat. Commun..

[53] Laitinen T., Ollikainen V., Lazaro C., Kauppi P., de Cid R., Anto J.M., Estivill X., Lokki H., Mannila H., Laitinen L.A. (2000). Association study of the chromosomal region containing the FCER2 gene suggests it has a regulatory role in atopic disorders. Am. J. Respir. Crit. Care Med..

[54] Koster E.S., Maitland-van der Zee A.H., Tavendale R., Mukhopadhyay S., Vijverberg S.J., Raaijmakers J.A., Palmer C.N. (2011). FCER2 T2206C variant associated with chronic symptoms and exacerbations in steroid-treated asthmatic children. Allergy.

[55] Stanic B., van de Veen W., Wirz O.F., Ruckert B., Morita H., Sollner S., Akdis C.A., Akdis M. (2015). IL-10-overexpressing B cells regulate innate and adaptive immune responses. J. Allergy Clin. Immunol.

[56] Uhm T.G., Kim B.S., Chung I.Y. (2012). Eosinophil development, regulation of eosinophil-specific genes, and role of eosinophils in the pathogenesis of asthma. Allergy Asthma Immunol. Res..

[57] Shirakawa I., Deichmann K.A., Izuhara I., Mao I., Adra C.N., Hopkin J.M. (2000). Atopy and asthma: Genetic variants of IL-4 and IL-13 signalling. Immunol. Today.

[58] Kabesch M., Depner M., Dahmen I., Weiland S.K., Vogelberg C., Niggemann B., Lau S., Illig T., Klopp N., Wahn U. (2007). Polymorphisms in eosinophil pathway genes, asthma and atopy. Allergy.

[59] Waage J., Standl M., Curtin J.A., Jessen L.E., Thorsen J., Tian C., Schoettler N., Flores C., The 23andMe Research Team, AAGC collaborators (2018). Genome-wide association and HLA fine-mapping studies identify risk loci and genetic pathways underlying allergic rhinitis. Nat. Genet..

[60] Blumenthal M., Marcus-Bagley D., Awdeh Z., Johnson B., Yunis E.J., Alper C.A. (1992). HLA-DR2, [HLA-B7, SC31, DR2], and [HLA-B8, SC01, DR3] haplotypes distinguish subjects with asthma from those with rhinitis only in ragweed pollen allergy. J. Immunol..

[61] Andiappan A.K., de Wang Y., Anantharaman R., Parate P.N., Suri B.K., Low H.Q., Li Y., Zhao W., Castagnoli P., Liu J. (2011). Genome-wide association study for atopy and allergic rhinitis in a Singapore Chinese population. PLoS ONE.

[62] Campbell A., Chanal I., Czarlewski W., Michel F.B., Bousquet J. (1997). Reduction of soluble ICAM-1 levels in nasal secretion by H1-blockers in seasonal allergic rhinitis. Allergy.

[63] Wei X., Zhang Y., Fu Z., Zhang L. (2013). The association between polymorphisms in the MRPL4 and TNF-α genes and susceptibility to allergic rhinitis. PLoS ONE.

[64] Ezell S.A., Polytarchou C., Hatziapostolou M., Guo A., Sanidas I., Bihani T., Comb M.J., Sourvinos G., Tsichlis P.N. (2012). The protein kinase Akt1 regulates the interferon response through phosphorylation of the transcriptional repressor EMSY. Proc. Natl. Acad. Sci. USA.

[65] Stockis J., Colau D., Coulie P.G., Lucas S. (2009). Membrane protein GARP is a receptor for latent TGF-β on the surface of activated human Treg. Eur. J. Immunol..

[66] Paternoster L., Standl M., Waage J., Baurecht H., Hotze M., Strachan D.P., Curtin J.A., Bonnelykke K., Tian C., Takahashi A. (2015). Multi-ancestry genome-wide association study of 21,000 cases and 95,000 controls identifies new risk loci for atopic dermatitis. Nat. Genet..

[67] Puel A., Ziegler S.F., Buckley R.H., Leonard W.J. (1998). Defective IL7R expression in T(-)B(+)NK(+) severe combined immunodeficiency. Nat. Genet..

[68] Gudbjartsson D.F., Bjornsdottir U.S., Halapi E., Helgadottir A., Sulem P., Jonsdottir G.M., Thorleifsson G., Helgadottir H., Steinthorsdottir V., Stefansson H. (2009). Sequence variants affecting eosinophil numbers associate with asthma and myocardial infarction. Nat. Genet..

[69] Mori T., Iwasaki Y., Seki Y., Iseki M., Katayama H., Yamamoto K., Takatsu K., Takaki S. (2014). Lnk/Sh2b3 controls the production and function of dendritic cells and regulates the induction of IFN-γ-producing T cells. J. Immunol..

[70] Sleiman P.M., Wang M.L., Cianferoni A., Aceves S., Gonsalves N., Nadeau K., Bredenoord A.J., Furuta G.T., Spergel J.M., Hakonarson H. (2014). GWAS identifies four novel eosinophilic esophagitis loci. Nat. Commun..

[71] Scott L.M., Civin C.I., Rorth P., Friedman A.D. (1992). A novel temporal expression pattern of three C/EBP family members in differentiating myelomonocytic cells. Blood.

[72] Gao H., Parkin S., Johnson P.F., Schwartz R.C. (2002). C/EBP γ has a stimulatory role on the IL-6 and IL-8 promoters. J. Biol. Chem..

[73] Leon B., Ballesteros-Tato A., Browning J.L., Dunn R., Randall T.D., Lund F.E. (2012). Regulation of T(H)2 development by CXCR5+ dendritic cells and lymphotoxin-expressing B cells. Nat. Immunol..

[74] Lawrence T. (2009). The nuclear factor NF-kappaB pathway in inflammation. Cold Spring Harb. Perspect. Biol..

[75] Shinnakasu R., Inoue T., Kometani K., Moriyama S., Adachi Y., Nakayama M., Takahashi Y., Fukuyama H., Okada T., Kurosaki T. (2016). Regulated selection of germinal-center cells into the memory B cell compartment. Nat. Immunol..

[76] Roychoudhuri R., Clever D., Li P., Wakabayashi Y., Quinn K.M., Klebanoff C.A., Ji Y., Sukumar M., Eil R.L., Yu Z. (2016). BACH2 regulates CD8(+) T cell differentiation by controlling access of AP-1 factors to enhancers. Nat. Immunol..

[77] Rothlin C.V., Ghosh S., Zuniga E.I., Oldstone M.B., Lemke G. (2007). TAM receptors are pleiotropic inhibitors of the innate immune response. Cell.

[78] Kassmeier M.D., Mondal K., Palmer V.L., Raval P., Kumar S., Perry G.A., Anderson D.K., Ciborowski P., Jackson S., Xiong Y. (2012). VprBP binds full-length RAG1 and is required for B-cell development and V(D)J recombination fidelity. EMBO J..

[79] Hamblet C.E., Makowski S.L., Tritapoe J.M., Pomerantz J.L. (2016). NK Cell Maturation and Cytotoxicity Are Controlled by the Intramembrane Aspartyl Protease SPPL3. J. Immunol..

[80] Andersen J.B., Strandbygard D.J., Hartmann R., Justesen J. (2004). Interaction between the 2′-5′ oligoadenylate synthetase-like protein p59 OASL and the transcriptional repressor methyl CpG-binding protein 1. Eur. J. Biochem..

[81] Anderson D.M., Maraskovsky E., Billingsley W.L., Dougall W.C., Tometsko M.E., Roux E.R., Teepe M.C., DuBose R.F., Cosman D., Galibert L. (1997). A homologue of the TNF receptor and its ligand enhance T-cell growth and dendritic-cell function. Nature.

[82] Mak A.C.Y., White M.J., Eckalbar W.L., Szpiech Z.A., Oh S.S., Pino-Yanes M., Hu D., Goddard P., Huntsman S., Galanter J. (2018). Whole-Genome Sequencing of Pharmacogenetic Drug Response in Racially Diverse Children with Asthma. Am. J. Respir. Crit Care Med..

[83] Galata G., Garcia-Montero A.C., Kristensen T., Dawoud A.A.Z., Munoz-Gonzalez J.I., Meggendorfer M., Guglielmelli P., Hoade Y., Alvarez-Twose I., Gieger C. (2021). Genome-wide association study identifies novel susceptibility loci for KIT D816V positive mastocytosis. Am. J. Hum. Genet..

[84] Ramasamy A., Kuokkanen M., Vedantam S., Gajdos Z.K., Couto Alves A., Lyon H.N., Ferreira M.A., Strachan D.P., Zhao J.H., Abramson M.J. (2012). Genome-wide association studies of asthma in population-based cohorts confirm known and suggested loci and identify an additional association near HLA. PLoS ONE.

[85] Portelli M.A., Hodge E., Sayers I. (2015). Genetic risk factors for the development of allergic disease identified by genome-wide association. Clin. Exp. Allergy.

[86] Wang Y., Lv L., Zang H., Gao Z., Zhang F., Wang X., Zhou X. (2015). Regulation of Trek1 expression in nasal mucosa with allergic rhinitis by specific immunotherapy. Cell Biochem. Funct..

[87] Jiang J., Liu J.Q., Li J., Li M., Chen H.B., Yan H., Mo L.H., Qiu S.Q., Liu Z.G., Yang P.C. (2015). Trek1 contributes to maintaining nasal epithelial barrier integrity. Sci. Rep..

[88] Cho J.S., Kang J.H., Han I.H., Um J.Y., Park I.H., Lee S.H., Lee H.M. (2015). Antiallergic Effects of Trichostatin A in a Murine Model of Allergic Rhinitis. Clin. Exp. Otorhinolaryngol..

[89] Zhu H., Shan L., Schiller P.W., Mai A., Peng T. (2010). Histone deacetylase-3 activation promotes tumor necrosis factor-α (TNF-α) expression in cardiomyocytes during lipopolysaccharide stimulation. J. Biol. Chem..

[90] Stefanowicz D., Hackett T.L., Garmaroudi F.S., Gunther O.P., Neumann S., Sutanto E.N., Ling K.M., Kobor M.S., Kicic A., Stick S.M. (2012). DNA methylation profiles of airway epithelial cells and PBMCs from healthy, atopic and asthmatic children. PLoS ONE.

[91] Swamy R.S., Reshamwala N., Hunter T., Vissamsetti S., Santos C.B., Baroody F.M., Hwang P.H., Hoyte E.G., Garcia M.A., Nadeau K.C. (2012). Epigenetic modifications and improved regulatory T-cell function in subjects undergoing dual sublingual immunotherapy. J. Allergy Clin. Immunol..

[92] Bayrak Degirmenci P., Aksun S., Altin Z., Bilgir F., Arslan I.B., Colak H., Ural B., Solakoglu Kahraman D., Diniz G., Ozdemir B. (2018). Allergic Rhinitis and Its Relationship with IL-10, IL-17, TGF-β, IFN-γ, IL 22, and IL-35. Dis. Markers.

[93] Li J.Y., Zhang Y., Lin X.P., Ruan Y., Wang Y., Wang C.S., Zhang L. (2016). Association between DNA hypomethylation at IL13 gene and allergic rhinitis in house dust mite-sensitized subjects. Clin. Exp. Allergy.

[94] Sarnowski C., Laprise C., Malerba G., Moffatt M.F., Dizier M.H., Morin A., Vincent Q.B., Rohde K., Esparza-Gordillo J., Margaritte-Jeannin P. (2016). DNA methylation within melatonin receptor 1A (MTNR1A) mediates paternally transmitted genetic variant effect on asthma plus rhinitis. J. Allergy Clin. Immunol..

[95] Piletic K., Kunej T. (2016). MicroRNA epigenetic signatures in human disease. Arch. Toxicol..

[96] Liu W., Ouyang H., Zeng Q., Luo R., Lu G. (2019). Decreased Treg-derived miR-181a and miR-155 correlated with reduced number and function of Treg cells in allergic rhinitis children. Eur. Arch. Otorhinolaryngol..

[97] Chen R.F., Huang H.C., Ou C.Y., Hsu T.Y., Chuang H., Chang J.C., Wang L., Kuo H.C., Yang K.D. (2010). MicroRNA-21 expression in neonatal blood associated with antenatal immunoglobulin E production and development of allergic rhinitis. Clin. Exp. Allergy.

[98] Liu W., Zeng Q., Luo R. (2016). Correlation between Serum Osteopontin and miR-181a Levels in Allergic Rhinitis Children. Mediat. Inflamm..

[99] Suojalehto H., Lindstrom I., Majuri M.L., Mitts C., Karjalainen J., Wolff H., Alenius H. (2014). Altered microRNA expression of nasal mucosa in long-term asthma and allergic rhinitis. Int. Arch. Allergy Immunol..

[100] Fan Y., Tang Z., Sun J., Zhao X., Li Z., Zheng Y., Zeng X., Feng J. (2021). MicroRNA-29a promotes the proliferation of human nasal epithelial cells and inhibits their apoptosis and promotes the development of allergic rhinitis by down-regulating FOS expression. PLoS ONE.

[101] Panganiban R.P., Wang Y., Howrylak J., Chinchilli V.M., Craig T.J., August A., Ishmael F.T. (2016). Circulating microRNAs as biomarkers in patients with allergic rhinitis and asthma. J. Allergy Clin. Immunol..

[102] Korde A., Ahangari F., Haslip M., Zhang X., Liu Q., Cohn L., Gomez J.L., Chupp G., Pober J.S., Gonzalez A. (2020). An endothelial microRNA-1-regulated network controls eosinophil trafficking in asthma and chronic rhinosinusitis. J. Allergy Clin. Immunol..

[103] Yamada Y., Kosaka K., Miyazawa T., Kurata-Miura K., Yoshida T. (2014). miR-142-3p enhances FcepsilonRI-mediated degranulation in mast cells. Biochem. Biophys. Res. Commun..

[104] Mayoral R.J., Deho L., Rusca N., Bartonicek N., Saini H.K., Enright A.J., Monticelli S. (2011). MiR-221 influences effector functions and actin cytoskeleton in mast cells. PLoS ONE.

[105] Song J., Ouyang Y., Che J., Li X., Zhao Y., Yang K., Zhao X., Chen Y., Fan C., Yuan W. (2017). Potential Value of miR-221/222 as Diagnostic, Prognostic, and Therapeutic Biomarkers for Diseases. Front. Immunol..

[106] Ishizaki T., Tamiya T., Taniguchi K., Morita R., Kato R., Okamoto F., Saeki K., Nomura M., Nojima Y., Yoshimura A. (2011). miR126 positively regulates mast cell proliferation and cytokine production through suppressing Spred1. Genes Cells.

[107] Haj-Salem I., Fakhfakh R., Berube J.C., Jacques E., Plante S., Simard M.J., Bosse Y., Chakir J. (2015). MicroRNA-19a enhances proliferation of bronchial epithelial cells by targeting TGFbetaR2 gene in severe asthma. Allergy.

[108] Caserta S., Kern F., Cohen J., Drage S., Newbury S.F., Llewelyn M.J. (2016). Circulating Plasma microRNAs can differentiate Human Sepsis and Systemic Inflammatory Response Syndrome (SIRS). Sci. Rep..

[109] Kumar M., Ahmad T., Sharma A., Mabalirajan U., Kulshreshtha A., Agrawal A., Ghosh B. (2011). Let-7 microRNA-mediated regulation of IL-13 and allergic airway inflammation. J. Allergy Clin. Immunol..

[110] Li L., Zhang S., Jiang X., Liu Y., Liu K., Yang C. (2018). MicroRNA-let-7e regulates the progression and development of allergic rhinitis by targeting suppressor of cytokine signaling 4 and activating Janus kinase 1/signal transducer and activator of transcription 3 pathway. Exp. Ther. Med..

[111] Gao Y., Yu Z. (2018). MicroRNA16 inhibits interleukin13induced inflammatory cytokine secretion and mucus production in nasal epithelial cells by suppressing the IkappaB kinase β/nuclear factorkappaB pathway. Mol. Med. Rep..

[112] Shaoqing Y., Ruxin Z., Guojun L., Zhiqiang Y., Hua H., Shudong Y., Jie Z. (2011). Microarray analysis of differentially expressed microRNAs in allergic rhinitis. Am. J. Rhinol. Allergy.

[113] Kohlhaas S., Garden O.A., Scudamore C., Turner M., Okkenhaug K., Vigorito E. (2009). Cutting edge: The Foxp3 target miR-155 contributes to the development of regulatory T cells. J. Immunol..

[114] Rodriguez A., Vigorito E., Clare S., Warren M.V., Couttet P., Soond D.R., van Dongen S., Grocock R.J., Das P.P., Miska E.A. (2007). Requirement of bic/microRNA-155 for normal immune function. Science.

[115] Martinez-Nunez R.T., Louafi F., Sanchez-Elsner T. (2011). The interleukin 13 (IL-13) pathway in human macrophages is modulated by microRNA-155 via direct targeting of interleukin 13 receptor alpha1 (IL13Ralpha1). J. Biol. Chem..

[116] Suojalehto H., Toskala E., Kilpelainen M., Majuri M.L., Mitts C., Lindstrom I., Puustinen A., PLoSila T., Sipila J., Wolff H. (2013). MicroRNA profiles in nasal mucosa of patients with allergic and nonallergic rhinitis and asthma. Int. Forum Allergy Rhinol..

[117] Jia M., Chu C., Wang M. (2018). Correlation of microRNA profiles with disease risk and severity of allergic rhinitis. Int. J. Clin. Exp. Pathol..

[118] Wang T., Chen D., Wang P., Xu Z., Li Y. (2018). miR-375 prevents nasal mucosa cells from apoptosis and ameliorates allergic rhinitis via inhibiting JAK2/STAT3 pathway. Biomed. Pharmacother..

[119] Deng Y.Q., Yang Y.Q., Wang S.B., Li F., Liu M.Z., Hua Q.Q., Tao Z.Z. (2015). Intranasal Administration of Lentiviral miR-135a Regulates Mast Cell and Allergen-Induced Inflammation by Targeting GATA-3. PLoS ONE.

[120] May J.R., Dolen W.K. (2017). Management of Allergic Rhinitis: A Review for the Community Pharmacist. Clin. Ther..

[121] Oktemer T., Altintoprak N., Muluk N.B., Senturk M., Kar M., Bafaqeeh S.A., Bellussi L., Passali D., Cingi C. (2016). Clinical efficacy of immunotherapy in allergic rhinitis. Am. J. Rhinol. Allergy.

[122] Karakoc-Aydiner E., Eifan A.O., Baris S., Gunay E., Akturk E., Akkoc T., Bahceciler N.N., Barlan I.B. (2015). Long-Term Effect of Sublingual and Subcutaneous Immunotherapy in Dust Mite-Allergic Children with Asthma/Rhinitis: A 3-Year Prospective Randomized Controlled Trial. J. Investig. Allergol. Clin. Immunol..

[123] Scadding G.W., Calderon M.A., Shamji M.H., Eifan A.O., Penagos M., Dumitru F., Sever M.L., Bahnson H.T., Lawson K., Harris K.M. (2017). Effect of 2 Years of Treatment with Sublingual Grass Pollen Immunotherapy on Nasal Response to Allergen Challenge at 3 Years Among Patients With Moderate to Severe Seasonal Allergic Rhinitis: The GRASS Randomized Clinical Trial. JAMA.

[124] Kamekura R., Yamashita K., Jitsukawa S., Nagaya T., Ito F., Ichimiya S., Himi T. (2016). Role of Crosstalk between Epithelial and Immune Cells, the Epimmunome, in Allergic Rhinitis Pathogenesis. Adv. Otorhinolaryngol..

[125] Potaczek D.P., Harb H., Michel S., Alhamwe B.A., Renz H., Tost J. (2017). Epigenetics and allergy: From basic mechanisms to clinical applications. Epigenomics.

[126] Schaafsma W., Zhang X., van Zomeren K.C., Jacobs S., Georgieva P.B., Wolf S.A., Kettenmann H., Janova H., Saiepour N., Hanisch U.K. (2015). Long-lasting pro-inflammatory suppression of microglia by LPS-preconditioning is mediated by RelB-dependent epigenetic silencing. Brain Behav. Immun..

[127] Xiang R., Liu Y., Xu Y. (2016). Effect of the FOXP3 gene methylation status in pathogenesis of patients with allergic rhinitis. Lin Chung Er Bi Yan Hou Tou Jing Wai Ke Za Zhi.

[128] Huehn J., Polansky J.K., Hamann A. (2009). Epigenetic control of FOXP3 expression: The key to a stable regulatory T-cell lineage?. Nat. Rev. Immunol..

[129] Toker A., Engelbert D., Garg G., Polansky J.K., Floess S., Miyao T., Baron U., Duber S., Geffers R., Giehr P. (2013). Active demethylation of the Foxp3 locus leads to the generation of stable regulatory T cells within the thymus. J. Immunol..

[130] Liu H.J., Zhang A.F., Zhao N., Li X.Z. (2016). Role of miR-146a in Enforcing Effect of Specific Immunotherapy on Allergic Rhinitis. Immunol. Investig..

[131] Durham A., Chou P.C., Kirkham P., Adcock I.M. (2010). Epigenetics in asthma and other inflammatory lung diseases. Epigenomics.

[132] Cheng R.Y., Shang Y., Limjunyawong N., Dao T., Das S., Rabold R., Sham J.S., Mitzner W., Tang W.Y. (2014). Alterations of the lung methylome in allergic airway hyper-responsiveness. Environ. Mol. Mutagen..

[133] Anna A., Monika G. (2018). Splicing mutations in human genetic disorders: Examples, detection, and confirmation. J. Appl. Genet..

[134] Xue H., Li J., Xie H., Wang Y. (2018). Review of Drug Repositioning Approaches and Resources. Int. J. Biol. Sci..

[135] Nelson M.R., Tipney H., Painter J.L., Shen J., Nicoletti P., Shen Y., Floratos A., Sham P.C., Li M.J., Wang J. (2015). The support of human genetic evidence for approved drug indications. Nat. Genet..

[136] Smith D., Helgason H., Sulem P., Bjornsdottir U.S., Lim A.C., Sveinbjornsson G., Hasegawa H., Brown M., Ketchem R.R., Gavala M. (2017). A rare IL33 loss-of-function mutation reduces blood eosinophil counts and protects from asthma. PLoS Genet..

[137] Mills M.C., Rahal C. (2019). A scientometric review of genome-wide association studies. Commun. Biol..

[138] Pinart M., Benet M., Annesi-Maesano I., von Berg A., Berdel D., Carlsen K.C., Carlsen K.H., Bindslev-Jensen C., Eller E., Fantini M.P. (2014). Comorbidity of eczema, rhinitis, and asthma in IgE-sensitised and non-IgE-sensitised children in MeDALL: A population-based cohort study. Lancet Respir. Med..

[139] Kaminsky D.A. (2014). Systems biology approach for subtyping asthma; where do we stand now?. Curr. Opin. Pulm. Med..

[140] Mc Mahon S.S., Sim A., Filippi S., Johnson R., Liepe J., Smith D., Stumpf M.P. (2014). Information theory and signal transduction systems: From molecular information processing to network inference. Semin. Cell Dev. Biol..

[141] Greene C.S., Tan J., Ung M., Moore J.H., Cheng C. (2014). Big data bioinformatics. J. Cell Physiol..

[142] Barnes K.C. (2010). Genomewide association studies in allergy and the influence of ethnicity. Curr. Opin. Allergy Clin. Immunol..

